# l-Arginine Induces White Adipose Tissue Browning—A New Pharmaceutical Alternative to Cold

**DOI:** 10.3390/pharmaceutics14071368

**Published:** 2022-06-28

**Authors:** Andjelika Kalezic, Aleksandra Korac, Bato Korac, Aleksandra Jankovic

**Affiliations:** 1 Institute for Biological Research “Sinisa Stankovic”—National Institute of Republic of Serbia, University of Belgrade, 11060 Belgrade, Serbia; andjelika.kalezic@ibiss.bg.ac.rs (A.K.); koracb@ibiss.bg.ac.rs (B.K.); 2 Faculty of Biology, Center for Electron Microscopy, University of Belgrade, 11060 Belgrade, Serbia; aleksandra.korac@bio.bg.ac.rs

**Keywords:** nitric oxide, l-arginine, browning, obesity

## Abstract

The beneficial effects of l-arginine supplementation in obesity and type II diabetes involve white adipose tissue (WAT) reduction and increased substrate oxidation. We aimed to test the potential of l-arginine to induce WAT browning. Therefore, the molecular basis of browning was investigated in retroperitoneal WAT (rpWAT) of rats exposed to cold or treated with 2.25% l-arginine for 1, 3, and 7 days. Compared to untreated control, levels of inducible nitric oxide (NO) synthase protein expression and NO signaling increased in both cold-exposed and l-arginine-treated groups. These increases coincided with the appearance of multilocular adipocytes and increased expression levels of uncoupling protein 1 (UCP1), thermogenic and beige adipocyte-specific genes (*Cidea*, *Cd137*, and *Tmem26*), mitochondriogenesis markers (peroxisome proliferator-activated receptor (PPAR)-γ coactivator-1α, mitochondrial DNA copy number), nuclear respiratory factor 1, PPARα and their respective downstream lipid oxidation enzymes after l-arginine treatment. Such browning phenotype in the l-arginine-treated group was concordant with end-course decreases in leptinaemia, rpWAT mass, and body weight. In conclusion, l-arginine mimics cold-mediated increases in NO signaling in rpWAT and induces molecular and structural fingerprints of rpWAT browning. The results endorse l-arginine as a pharmaceutical alternative to cold exposure, which could be of great interest in obesity and associated metabolic diseases.

## 1. Introduction

An imbalance in energy intake and expenditure leads to an unhealthy surplus of white adipose tissue (WAT), i.e., obesity, which represents a fertile ground for various metabolic diseases. Accumulating evidence demonstrates that a shift from WAT toward brown adipose tissue (BAT)-like phenotype, via so-called browning, may restrain obesity and improve overall metabolic profile (as reviewed recently in [1]). Namely, genuine brown and beige adipocytes that appear during WAT browning upon stimulation attain a unique capacity to burn lipids and dissipate chemical energy as heat via uncoupled respiration. WAT browning is enabled by structural and metabolic reprogramming, including lipid body remodeling from unilocular to multilocular morphology, mitochondriogenesis, and an upsurge in the expression of fatty acid oxidation enzymes and ultimately uncoupling protein 1 (UCP1) [2]. Activation of UCP1 in interscapular BAT increases the uptake and oxidation of metabolic substrates to boost the adaptive non-shivering thermogenesis [3,4]. Increasing such functional, BAT-like features in WAT may help in the weight-gain regulation [5] and in buffering excess nutrients and potentially noxious metabolites [6,7]. Therefore, WAT browning is an attractive therapeutic target for obesity and associated metabolic diseases. Hypermetabolic states (such as cold exposure and exercise) and various nutritional and pharmacological agents can induce WAT browning (as reviewed in [8]); however, there is a lack of safe and efficient browning inducers that could be therapeutically exploited. 

Cold-induced β-adrenergic signaling is the primary physiological inducer of thermogenesis in BAT and browning in WAT [8]. The thermogenic response of BAT is mediated by increased levels of redox-active molecule nitric oxide (NO) [9]. Two out of three nitric oxide synthase (NOS) isoforms contribute to NO synthesis in adipose tissues, endothelial and inducible NOS (eNOS and iNOS, respectively) [10]. Both isoforms are sensitive to sympathetic signaling in BAT, contributing to NO production upon cold exposure [11,12] and mediating and amplifying adaptive thermogenesis in BAT. Namely, NO signaling acts on the transcription program and metabolic reprogramming, supporting the processes involved in large-scale tissue remodeling (angiogenesis, vascularisation, proliferation, and differentiation) [9,13]. In WAT, inorganic nitrite supplementation has been shown to activate browning by increasing NO levels [14,15]. Several studies indicated that this non-canonical, reductive pathway of NO production, which is attributed to distinct enzymes possessing nitrite reductase activity, occurs in physiologic conditions, confined to specific acidic or hypoxic conditions [16]. On the other hand, the involvement of the NOS/NO pathway—the canonical NO synthesis pathway [17] through oxidative degradation of l-arginine to citrulline catalyzed by NOS isoforms—in WAT browning remains unclear.

When applied in vivo, a substrate for NOS-mediated NO synthesis, l-arginine, increases the rate of endogenous NO synthesis and cGMP-dependent signaling [18]. Besides, NO may act via nitrosation- and nitration-mediated posttranslational redox regulatory mechanisms that affect gene and protein expression, enzyme activity, cell morphology, and tissue structure [19]. Previous studies have shown that l-arginine has beneficial effects on metabolic homeostasis, acting on thermogenesis in BAT and overall oxidative metabolism, resulting in fat mass reduction while sparing lean body mass in both animal and human studies [18,20,21,22,23,24,25,26,27,28,29]. However, whether such beneficial effects of l-arginine involve WAT browning is currently unknown.

Therefore, in the present study, we aimed to examine the involvement of the NOS/NO synthesis pathway in rpWAT browning during cold exposure and whether a priory upregulation of this pathway by a physiological dose of l-arginine instigates the browning process. Therefore, we examined the effects of l-arginine on NO levels and key enzymes involved in the l-arginine/NO synthesis pathway during 1, 3, and 7 days of cold exposure and 2.25% l-arginine treatment. Additionally, we assessed structural, metabolic, and molecular indicators of rpWAT browning, including nuclear respiratory factor 1 (NRF1), peroxisome proliferator-activated receptor α (PPARα), peroxisome proliferator-activated receptor-gamma coactivator-1α (PGC-1α), mitochondrial DNA copy number (MCN), enzymes involved in fatty acid turnover and oxidation, thermogenic and beige adipocyte-specific genes and the pattern of UCP1 expression in parallel with the occurrence of multilocular adipocytes morphology. The results indicate that the NOS/NO pathway is involved in cold-mediated rpWAT browning and strongly suggest that l-arginine could be an effective pharmaceutical tool for the induction of WAT browning.

## 2. Materials and Methods

### 2.1. Animal Study

All common chemicals such as digestion enzymes, antibiotics, and buffers were obtained from Sigma-Aldrich (Sigma-Aldrich Chemie GmbH, Munich, Germany), unless stated otherwise. First, 2.5 months old, age- and weight-matched male Mill Hill hooded rats (*Rattus norvegicus*, Berkenhout, 1769) raised in the vivarium at the Institute for Biological Research “Sinisa Stankovic” were randomly divided into three groups (*n* = 6): control (maintained at 24 ± 1 °C), cold-exposed (maintained in a cold chamber at 4 ± 1 °C for 1, 3, or 7 days), and l-arginine-treated (ad libitum 2.25% solution of l-arginine ∙HCl for 1, 3, or 7 days). Animals were housed in conventional cages under a 12 h light/dark photoperiod with free access to standard chow food and water ad libitum. Food and water intake were monitored daily. Following cold exposure and l-arginine treatment, body weights were measured, animals were sacrificed by decapitation, and visceral white adipose tissues (WAT) depots, including retroperitoneal (rpWAT), mesenteric (mWAT), and epididymal (eWAT), were immediately dissected and weighted. rpWAT was further processed for molecular, microscopy, and ex vivo analyses. Adiposity index was calculated as the quotient of the sum of the rpWAT, mWAT, and gWAT mass (g) and total body weight (g) × 100. Blood was collected, allowed to clot, and centrifuged at 3500× *g* to obtain serum.

### 2.2. Biochemistry/ELISA Assays

Blood glucose was measured following decapitation by glucose-oxidase reagent strips (GlucoSure test, Prizma, Kragujevac, Serbia). Serum lipid parameters (total cholesterol, LDL-cholesterol, HDL-cholesterol, and triglycerides) were determined using standard biochemical methods on an ILab300+ analyzer (Instrumentation Laboratory, Milan, Italy). All reagents used for biochemical analyses were purchased from BioSystems (Barcelona, Spain). Leptin and adiponectin levels were determined by Rat Leptin and Adiponectin ELISA kits (KRC2281, Invitrogen Thermo Fisher Scientific, Waltham, MA, USA and E-EL-R3012, Elabscience, Huston, TX, USA), according to the manufacturer’s instructions.

### 2.3. Isolation of Mature Adipocytes

For mature adipocytes isolation and culture, rats were kept for 7 days either untreated (controls), exposed to cold (4 ± 1 °C), or treated with l-arginine (ad libitum 2.25% solution of l-arginine ∙HCl). After decapitation, whole rpWAT depots were weighed and placed into capped, sterile 50 mL conical tubes containing sterile transport medium (Dulbecco phosphate-buffered saline, pH 7.4 with 1% penicillin-streptomycin). Tissues were transported to the laminar flow hood within 5 min, transferred into petri dishes with sterile digestion buffer pre-warmed to 37 °C (Medium 199, 1% penicillin-streptomycin, 1 mg/mL collagenase Type II from *Clostridium histolyticum* and 1 mg/mL dispase II), and minced for approximately 2 min to the homogenous mixture. Three mL of digestion buffer were used per one gram of rpWAT. Finely minced adipose tissue was transferred to 50 mL tubes and left in the water bath at 37 °C for 35 min. Every 5 min, the stage of digestion was checked visually, and tubes were vortexed for 10 s. Following tissue digestion, undigested pieces of tissue were removed by filtration through a sterile gauze mesh, and adipocytes were resuspended and washed two times with 50 mL of wash buffer (M199 medium supplemented with 10% fetal bovine serum and 1% penicillin-streptomycin). Each time, adipocytes were allowed to float out for 3–5 min, and the infranatant containing the wash buffer was removed with an 18-gauge needle and syringe. Finally, mature adipocytes were centrifuged for 30 s at 800 rpm, and the infranatant and lipid layer above the floating adipocytes were carefully removed and discarded. To calculate the number of adipocytes, 20 μL of packed adipocytes were mixed with an equal volume of 0.4% trypan blue, and cells were counted using a hemocytometer chamber under a light microscope with 20× magnification. Isolated mature white adipocytes were used for further analyses.

### 2.4. NO Production

Measurements were done in triplicate per 100 μL of packed adipocytes (~6 × 10^5^ adipocytes). Production of NO by isolated mature adipocytes was measured electrochemically with a highly selective NO-sensitive electrode (amiNO-700 sensor electrode; Innovative Instruments Inc., Tampa, FL, USA). The sensor was polarized in an aqueous solution for at least 24 h before calibration, as suggested by the manufacturer. The sensor was calibrated by the conversion of NO_2_ to NO in an acidic solution containing potassium iodide. To increase the NO output, ~6 × 10^5^ adipocytes were incubated for 30 min at 37 °C with 400 µL of fresh M199 medium (containing 300 µM l-arginine and supplemented with 10 µM superoxide dismutase mimetic manganese (III) tetrakis 4-benzoic acid porphyrin monochloride (Cayman Chemical, MI, USA)) prior to NO measurement. After incubation, the cells were spun for 5 s, and the infranatant was immediately thereafter used for NO measurement. NO was measured by adding 300 μL of medium sample to 50 μL of H_2_O, which served as the baseline for the sensor.

### 2.5. H&E Staining

Immediately after dissection and washing, one part of rpWAT tissue was fixed in 4% paraformaldehyde and routinely processed for embedding. Paraffin-embedded 7–10 μm-thick sections of rpWAT tissue were routinely stained with hematoxylin and eosin, as described previously [2]. 

### 2.6. Immunohistochemistry of UCP1

Semi-fine (1 μm thick) sections were used for the detection of UCP1 by routine immunohistochemistry, as described previously [2], using primary antibodies against UCP1 (1 µg/mL; ab10983) and appropriate secondary antibodies (all purchased from Abcam, Cambridge, UK). Briefly, before incubation with primary antibodies, antigen retrieval was performed in citrate buffer in the microwave for 5 min. Sections were incubated with primary antibodies overnight at 4 °C and incubated with secondary antibodies for 2 h at room temperature. For immunodetection and visualization, a Dako LSAB Universal Kit (Dako Scientific, Glostrup, Denmark) was used. Finally, sections were mounted with DPX (Sigma-Aldrich, St. Louis, MO, USA). All sections were counterstained with hematoxylin and analyzed with an optical light microscope (Leica DMLB microscope, Leica Microsystems, Wetzlar, Austria). Negative controls were obtained by omitting the primary antibody.

### 2.7. RT-PCR/Gene Expression Analysis

Total rpWAT RNA was extracted with Trizol (Invitrogen, Life Technologies, Waltham, MA, USA), and first-strand cDNA synthesis was done with a Revert Aid First Strand cDNA Synthesis Kit (Thermo Fischer Scientific, Waltham, MA, USA) according to the manufacturer’s instructions. The concentration and quality of RNA samples were assessed with a NanoPhotometer^®^ (Implen GmbH, München, Germany) at 260 nm and 280 nm. Real-time PCR was performed under standard conditions using a FastStart Essential DNA Green Master kit (Roche, Basel, Switzerland) and QuantStudio™ 3 ReaL-Time PCR System (Thermo Fischer Scientific, Waltham, MA, USA). Primer sequences and cycling conditions are given in Figure S1. As an internal standard for amplification, 18S was quantified. Prior optimization was conducted for each set of primers that consisted of optimal primer, MgCl_2_, template concentration determination, verification of amplification efficiency, and nonspecific amplification. PCR amplification was performed in duplicate in a total reaction volume of 10 μL.

### 2.8. Mitochondrial DNA Copy Number (MCN)

Total DNA was extracted with Trizol (Invitrogen, Life Technologies, Waltham, MA, USA), and the concentration was assessed with a NanoPhotometer^®^ (Implen GmbH, Munich, Germany) at 260 nm and 280 nm, subsequently, calculated per formula A_260_ of 1.0 = 50 µg/mL of pure double-stranded DNA. Then, 10 ng of total DNA were used for RT-PCR determination of relative mitochondrial copy number. RT-PCR was performed for the *18S* gene as the nuclear target and the *Nd4* gene as the mitochondrial target under the following cycling conditions: 95 °C for 10 min, 40 cycles at 95 °C for 15 s, 60 °C for 30 s, 72 °C for 45 s, and melt curve analysis at 95 °C for 15 s, 60 °C for 1 min, 95 °C for 1 s in a QuantStudio™ 3 ReaL-Time PCR System (Thermo Fischer Scientific, Waltham, MA, USA). Mitochondrial DNA content relative to nuclear DNA content was calculated as 2 × 2^ΔCT^, where ΔCT stands for CT_(18S)_–CT_(Nd4)_.

### 2.9. Western Blot/Protein Expression Analysis

Western blot analysis was conducted as described previously [2] using primary antibodies against endothelial nitric oxide synthase (eNOS, 1 µg/mL; sc-376751), inducible nitric oxide synthase (iNOS, 1 µg/mL; sc-8310), 3-nitrotyrosine (3-NT, 1 µg/mL; sc-65385), arginase I (1 µg/mL; sc-47715), arginase II (1 µg/mL; sc-393496), hormone sensitive lipase (HSL, 1 µg/mL; sc-74489), adipose triglyceride lipase (ATGL, 1 µg/mL; sc-365278), monoacylglycerol acyltransferase 1 (MGAT1, 1 µg/mL; sc-376079), carnitine palmitoyltransferase 1 (CPT1, 1 µg/mL; sc-393070), proliferating cell nuclear antigen (PCNA, 1 µg/mL; sc-7907), all purchased from Santa Cruz (Dallas, TX, USA), and nuclear factor erythroid 2-related factor 2 (Nrf2, 1 µg/mL; ab31163), uncoupling protein 1 (UCP1, 1 µg/mL; ab10983), β-actin (1 µg/mL; ab8226), nuclear respiratory factor 1 (NRF1, 1 µg/mL; ab86516), peroxisome proliferator-activated receptor-α (PPARα, 1 µg/mL; ab8934), acyL-CoA dehydrogenase medium chain (ACADM, 1 µg/mL; ab92461), all purchased from Abcam (Cambridge, UK). Immunoreactive bands were analyzed using the Image J software (Version v1.53c, National Institutes of Health, Bethesda, MD, USA). Band density for each protein of interest was normalized as the ratio of pixel intensity for the target protein averaged against the loading control (β-actin). The figures show the protein content as protein expression in arbitrary units (AU) from three independent experiments. Images of whole uncut blots are shown in Figure S1.

### 2.10. Statistical Analyses

All data were statistically analyzed using GraphPad Prism software (Version 8.4.3 GraphPad Software, San Diego, CA, USA). The Student’s *t*-test was used to evaluate differences between two groups, while one-way analysis of variance (ANOVA) followed by multiple comparison Dunnett’s post hoc test was used for evaluation of inter-group differences between multiple groups. Error bars represent standard deviation (SD) or standard error of the mean (SEM), as stated in the figure legends. Statistical significance was accepted at *p* < 0.05.

## 3. Results

### 3.1. Cold Exposure-Induced Browning Is Associated with Endogenous NO Synthesis; l-Arginine Increases NO Synthesis in rpWAT to a Similar Extent

**Figure 1:**
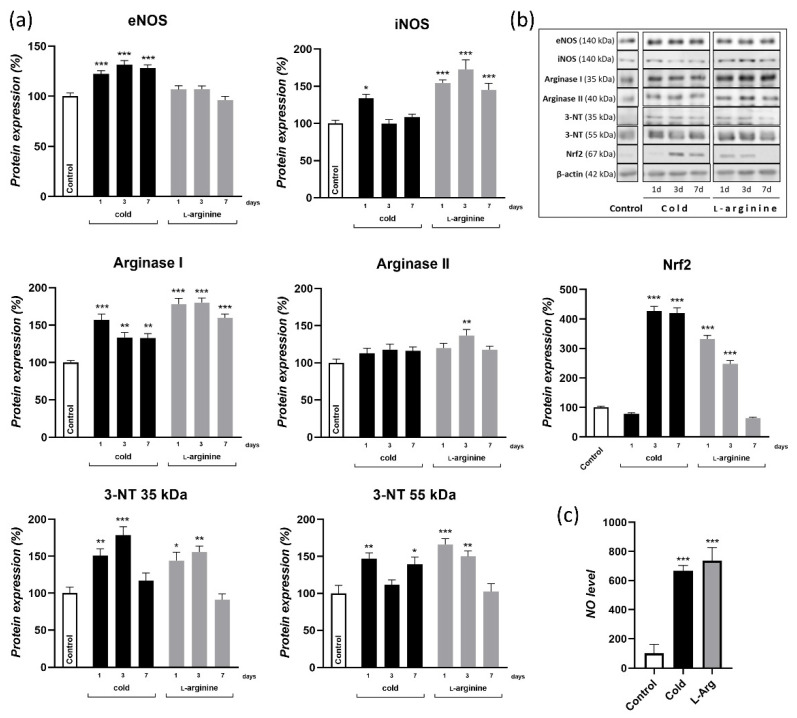
Induction of l-arginine/NO pathway in rpWAT of cold-exposed rats and the effects of 2.25% l-arginine supplementation. Protein expression analysis of endothelial NO synthase (eNOS), inducible NO synthase (iNOS), arginases I and II, nuclear factor-erythroid factor 2-related factor 2 (Nrf2), and 3-nitrotyrosine (3-NT)-modified proteins (represented by two-immunoreactive bands, at 35 and 55 KDa) during 1, 3, and 7 days of cold exposure and l-arginine treatment (**a**) and their corresponding blots (**b**). Each band is representative of six pooled samples per group. Protein content is expressed relative to control acclimated to room temperature (100%). Experiments were repeated in triplicate. Data were quantified as described in the Methods. (**c**) Detection of nitric oxide (NO) levels in the mature adipocytes stimulated by l-arginine (300 µM) for 30 min, at 37 °C, previously isolated from rpWAT of untreated (control), cold-exposed, and l-arginine (2.25%)-treated rats for 7 days (*n* = 9, 3 per group, in three technical replicates per group). Data represent the mean ± SEM. * Compared to control, * *p* < 0.05, ** *p* < 0.01 and *** *p* < 0.001.

Exposure to cold is one of the most powerful physiological stimuli for the induction of WAT browning. To examine if NO is involved in the cold-induced browning of rpWAT and whether the physiological dose of l-arginine increases the endogenous NO synthesis in rpWAT, we determined protein expression levels of enzymes involved in the arginine-NO synthesis pathway, 3-NT-modified proteins, and NO level during 7-day cold exposure and 2.25% l-arginine treatment. Two enzymes, eNOS and iNOS, that are present in WAT and metabolize l-arginine to NO and citrulline were examined. The results showed that, in comparison to room-temperature maintained controls, eNOS protein expression was increased following 1, 3, and 7 days of cold acclimation (*p* < 0.001). A more transient increase in iNOS protein expression was detected on day 1 of cold exposure (*p* < 0.05), which subsequently returned to the control level. During l-arginine supplementation, l-arginine predominately acted on iNOS protein expression in rpWAT, whose protein expression increased 50–75% compared to the control (Figure 1a). Adipocytes also express arginases, enzymes that restrain NOS-mediated NO synthesis, diverting l-arginine towards degradation to ornithine and urea. Protein expression of arginase I was significantly increased both following cold exposure and l-arginine treatment, while protein expression of arginase II was upregulated only on day 3 of l-arginine treatment (Figure 1a). The net result of NOSs and arginases induction was shifted toward higher NO synthesis, both during cold exposure and l-arginine treatment, since NO levels measured in adipocytes isolated from cold-exposed and l-arginine-treated rats were higher (*p* < 0.001) compared to adipocytes isolated from controls (Figure 1c). NO can be bound to heme proteins, including the soluble guanylate cyclase, increasing the cGMP synthesis and signaling, and/or can also propel downstream reactive nitrogen species (RNS) that act on various proteins via nitration- and nitrosation-related redox signaling. The expression pattern of 3-NT-modified proteins was examined in rpWAT, and the results have identified two bands (corresponding to 35 kD and 55 kD) whose intensity was increased both during cold exposure and, also, transiently on day 1 and day 3 of l-arginine treatment (Figure 1a). To assess the shift in the pro-nitrooxidative redox status, we additionally determined the expression of redox-rheostat—transcription factor nuclear factor-erythroid factor 2-related factor 2 (Nrf2) and found that its protein expression was increased in response to cold-induced and l-arginine-induced redox changes. Precisely, the protein level of Nrf2 increased following 3 and 7 days of cold exposure (*p* < 0.001) and following 1 and 3 days of l-arginine treatment (*p* < 0.001) (Figure 1c).

### 3.2. Effects of l-Arginine Treatment on Circulatory Parameters and Adipokines Levels

The effects of 7-day l-arginine treatment on several systemic circulating biochemical parameters and endocrine function of WAT are shown in Table 1. l-arginine treatment triggered an increase in the total, HDL-, and LDL-cholesterol levels, but these changes did not disturb the ratio between the cholesterols indicating atherogenic and heart disease risk. Besides, there was no difference in circulating glucose and triglyceride levels (measured in the fed state) between l-arginine-treated rats and controls, which is in line with equal food intake in examined groups. Serum adiponectin was not affected by l-arginine treatment, while serum leptin was decreased (*p* < 0.001).

### 3.3. Slimming Effects of l-Arginine Are Associated with Diminution of rpWAT Depot Mass

**Figure 2:**
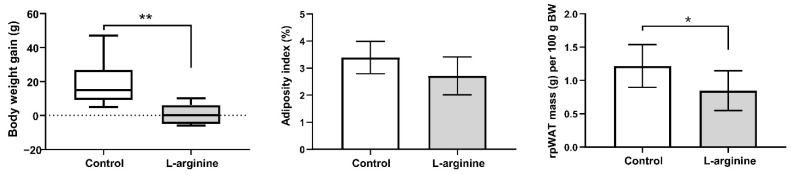
l-arginine reduces body weight gain and retroperitoneal white adipose tissue (rpWAT) mass. Body weight gain, adiposity index, and relative rpWAT mass of untreated (control) and l-arginine-treated rats; 2.5-month-old rats were randomly assigned to receive drinking water or 2.25% l-arginine∙HCl in drinking water for 7 days. * Comparison with control; (*n* = 6–8 per group); * *p* < 0.05; ** *p* < 0.01.

Body weight gain, adiposity index (the approximate index of intra-abdominal fat mass and cardiovascular risk), and relative rpWAT mass of l-arginine-treated animals and controls are presented in Figure 2. The treatment triggered a reduction in body weight gain (*p* < 0.01), which is associated with the lower relative mass of rpWAT (*p* < 0.05). Adiposity index was also determined following a 7-day l-arginine treatment and showed a reducing trend that did not reach statistical significance (*p* = 0.07).

### 3.4. Net Lipid Mobilization Effect of l-Arginine Is Associated with Increased Lipolysis

**Figure 3:**
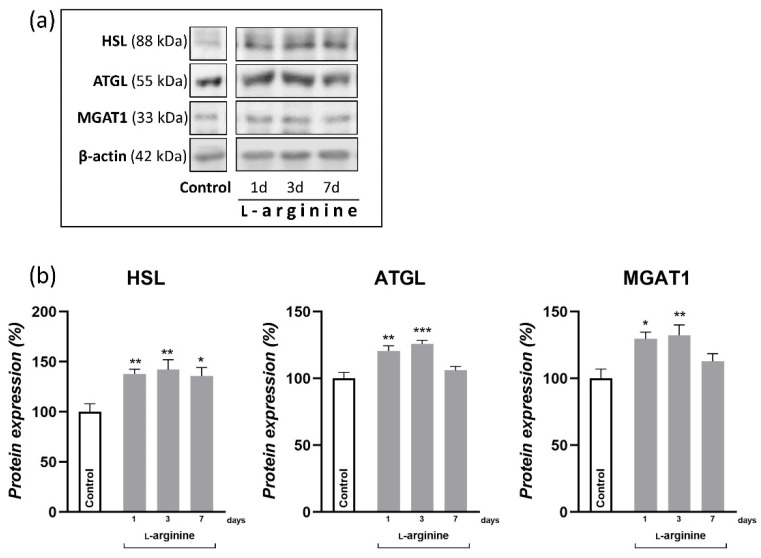
l-arginine increases fatty acid turnover in retroperitoneal white adipose tissue (rpWAT). Protein content of hormone sensitive lipase (HSL), adipose triglyceride lipase (ATGL), and monoacylglycerol O-acyltransferase 1 (MGAT1) in rpWAT of rats receiving either drinking water or 2.25% l-arginine∙HCl in drinking water for 1, 3, and 7 days, determined by Western blot. The signals from representative Western blots are shown (**a**). Data obtained after quantification of specific bands and expressed as % of the control group taken as 100% represent the mean ± SEM (**b**). Each band is representative of six pooled samples per group. Experiments were repeated in triplicate. Data were quantified as described in the Methods. * Comparison between control and l-arginine-treated group; * *p* < 0.05, ** *p* < 0.01, *** *p* < 0.001.

The relative mass of WAT is mainly determined by its lipid reserves. Namely, most of the adipocyte cell volume is occupied by triacylglycerols that are stored and mobilized via triacylglycerol synthesis and hydrolysis, respectively. Here, the expression levels of key enzymes involved in both processes are presented in Figure 3. Note that 1-day and 3-day l-arginine treatments increased the expression levels of hormone-sensitive lipase (HSL) and adipocyte triacylglycerol lipase (ATGL), in parallel with monoacylglycerol O-acyltransferase 1 (MGAT1). Following a 7-day long treatment, however, only HSL remained increased compared to the untreated control (*p* < 0.05).

### 3.5. l-Arginine Elicits an Increase in Expression of PGC-1α, NRF1, PPARα, and Their Downstream Mitochondrial Targets in rpWAT

**Figure 4:**
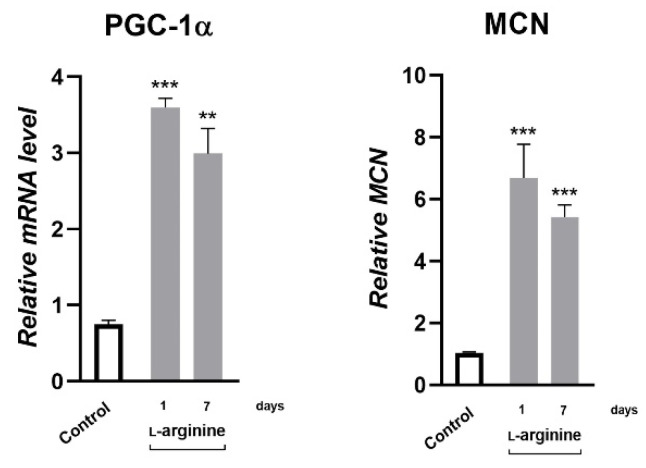
Peroxisome proliferator-activated receptor-gamma coactivator—1α (PGC-1α) gene expression and mitochondrial DNA copy number (MCN) level increase in retroperitoneal white adipose tissue (rpWAT) upon 1 and 7 days of 2.25% l-arginine∙HCl treatment. * Comparison with control (*n* = 6 per group); ** *p* < 0.01, *** *p* < 0.001.

**Figure 5:**
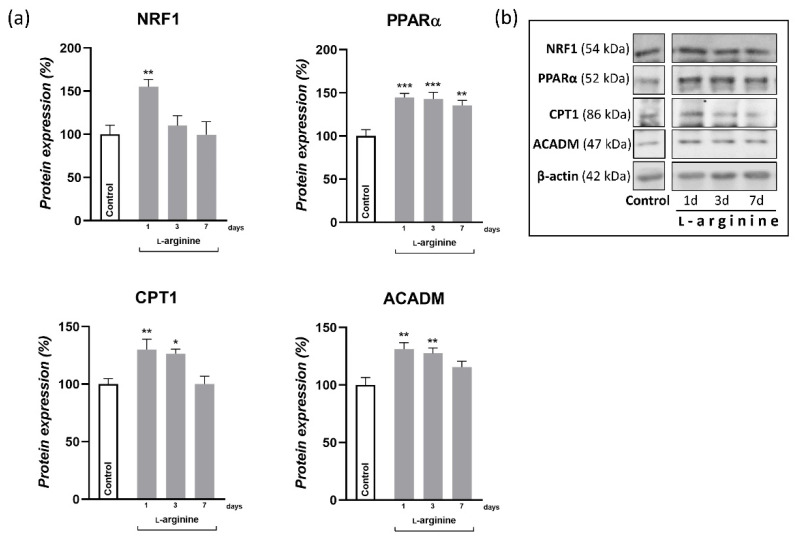
Protein content of nuclear respiratory factor 1 (NRF1), peroxisome proliferator-activated receptor-α (PPARα), carnitine palmitoyltransferase 1 (CPT1), and acyl-CoA dehydrogenase medium chain (ACADM) in retroperitoneal white adipose tissue (rpWAT) of rats receiving either drinking water or 2.25% l-arginine∙HCl in drinking water for 1, 3, and 7 days. Data obtained after quantification of specific bands and expressed as % of the control group taken as 100% represent the mean ± SEM (**a**). Each band is representative of six pooled samples per group (**b**). Experiments were repeated in triplicate. Data were quantified as described in the Methods. * Comparison between control and l-arginine-treated group; * *p* < 0.05, ** *p* < 0.01, *** *p* < 0.001.

Changes in response to l-arginine treatment at the level of PGC-1α gene expression and the protein expression of its downstream transcription targets, NRF1 and PPARα, as well as metabolic enzymes in rpWAT, are shown in Figure 4 and Figure 5, respectively. After 1 day of l-arginine treatment, PGC-1α mRNA expression increased significantly compared to the untreated control, and this increase was maintained until day 7 of l-arginine treatment. Such upregulation of PGC-1α was associated with a concordant increase in relative MCN (Figure 4) and the protein expression level of NRF1 and PPARα (Figure 5). In addition, a transient increase in the expression levels of enzymes that facilitate long-chain fatty acid transport*—*carnitine palmitoyl transferase 1 (CPT1) and subsequent β-oxidation*—*medium-chain acyl-CoA dehydrogenase (ACADM) in mitochondria were observed in rpWAT following l-arginine treatment (Figure 5). Interestingly, although the protein level of PPARα and the relative MCN remained increased on day 7 of treatment, the expression of NRF1 and ACADM returned to their control values on days 3 and 7 of the treatment, respectively (Figure 5).

### 3.6. UCP1 Protein Expression Level Increases upon l-Arginine Treatment and Over-Exceeds Cold-Induced Effects

**Figure 6:**
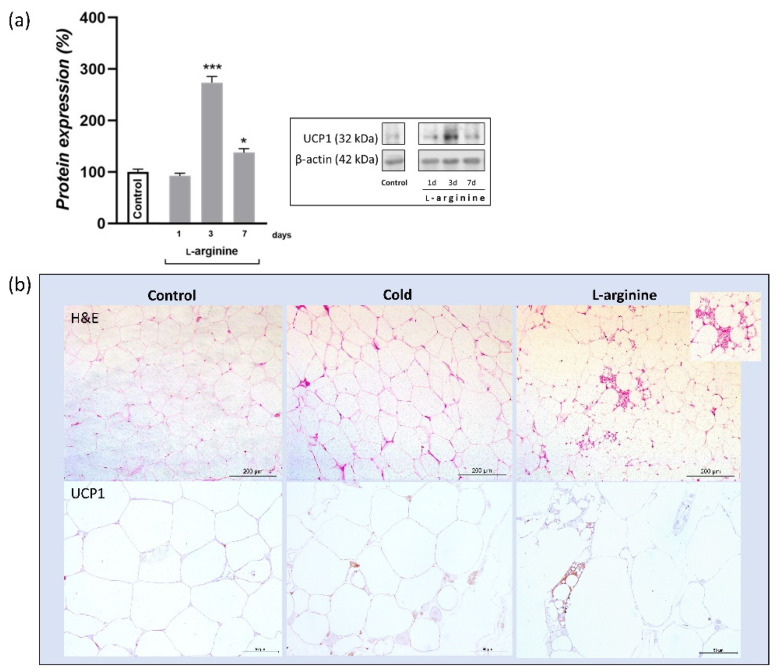
UCP1 protein expression level and tissue expression pattern in control, cold-exposed, and l-arginine-treated rats. Analysis of UCP1 protein expression after 1, 3, and 7 days of l-arginine treatment (**a**). Maximum induction of UCP1 was observed on day 3 of l-arginine treatment. Protein content is expressed relative to control rats (100%). Data represent the mean ± SEM. * Compared to control, * *p* < 0.05, *** *p* < 0.001. Experiments were repeated in triplicate. Data were quantified as described in the Methods. Analysis on the morphological level (H&E) indicates the simultaneous appearance of sporadic islets of multilocular cells selectively in retroperitoneal white adipose tissue (rpWAT) of l-arginine-treated rats (**b**). Inset, enlarged area of multilocular cells. Scale bars, 200 μm. Immunohistochemical staining for UCP1 (**b**, **lower panel**) showed that, compared with very weak UCP1-immunopositive adipocytes in the control, stronger UCP1-immunopositivity characterized adipocytes in rpWAT of rats exposed to cold and treated with l-arginine for 3 days. Moreover, highly positive UCP1-positive multilocular adipocytes appear only in the l-arginine-treated group of rats. Scale bars, 50 μm.

As compared to untreated control, protein expression of UCP1 was increased (2.8-fold, *p* < 0.001) following 3 days of l-arginine treatment; such an increase over-exceeds (91%) the cold exposure-induced increase in UCP1 protein level (data not shown) (Figure 6a). Additionally, islands of polygonal, multilocular adipocytes, strongly positive for UCP1, were observed only in L-arginine-treated rats (Figure 6b).

### 3.7. l-Arginine Elicits the Expression of Genetic Markers of Beige Adipocytes

**Figure 7:**
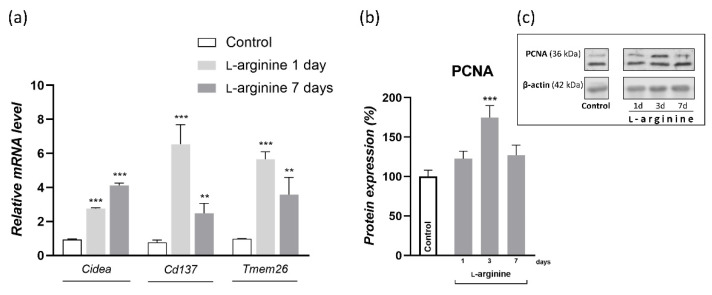
The l-arginine treatment induces gene expression of thermogenic beige adipocyte markers cell death-inducing DFFA like effector A (*Cidea*), *Tmem26*, and *Cd137* and protein levels of proliferation marker proliferating cell nuclear antigen (PCNA) in rat retroperitoneal white adipose tissue (rpWAT). Adipose tissue gene expression (mean ± SEM of normalized ratios with 18S) of *Cidea*, *Tmem26*, and *Cd137* in rats treated with 2.25% l-arginine∙HCl for 1 and 7 days is upregulated by l-arginine, as compared to the control group (**a**). Also, PCNA protein levels after 1, 3, and 7 days of l-arginine treatment were analyzed in rpWAT by Western blot, as presented in the Material and Methods section. The protein content is expressed relative to control rats (100%) (**b**), and the respective bands are presented (**c**). Data represent the mean ± SEM. * Compared to control, ** *p* < 0.01, *** *p* < 0.001. Experiments were repeated in triplicate.

To dissect the cellular basis of l-arginine-induced WAT browning, we investigated gene expression of thermogenic adipocytes marker (*Cidea*) and the more specific markers of beige adipocytes (*Cd137* and *Tmem26*) in rpWAT of l-arginine-treated animals and controls (Figure 7). After twenty-four hours of l-arginine treatment, gene expression levels of thermogenic and beige adipocyte markers, *Cidea*, *Cd137*, and *Tmem26* were all increased, and these levels were maintained until the end of the 7-day l-arginine treatment (Figure 7a). In parallel, protein expression of proliferation marker proliferating cell nuclear antigen (PCNA) increased on day 3 of l-arginine treatment (*p* < 0.001) (Figure 7b).

## 4. Discussion

The beneficial effects of l-arginine on metabolic homeostasis are mainly ascribed to its effects on fat mass reduction and energy metabolism, where WAT undergoes fat mobilization to meet the l-arginine-induced increase in energy metabolism in tissues with high oxidative capacity (primarily BAT and muscles). For the first time, the current study demonstrates that the beneficial effects of l-arginine can also be attributed to the ignition of WAT browning. Precisely, 7-day-l-arginine treatment at room temperature mirrored the 7-day-cold-induced increase in rpWAT NO levels. The increase concurred with the appearance of multilocular beige adipocytes, increased UCP1 protein expression, thermogenic and beige adipocyte-specific genetic markers expression, as well as the expression of their target transcription factors and metabolic enzymes involved in fatty acid turnover and mitochondrial β-oxidation. Finally, the end-course l-arginine treatment led to the decline in leptinaemia, rpWAT mass, and body weight. The results point to l-arginine as a pharmacological alternative to cold exposure for the induction of browning of WAT.

Low environmental temperature and sympathetic stimulation are prime factors regulating thermogenic recruitment of BAT and beige adipocytes in WAT depots, characterized primarily by an increase in the relative amount of UCP1 [2,30,31,32,33,34,35,36]. Upon cold exposure, eNOS and iNOS optimize NO-mediated vasodilatation [13,14] and recruit several processes underlining adaptive thermogenesis, including the UCP1 expression in BAT [15,25]. In the present study, we showed that protein expression of both eNOS and iNOS increased in rpWAT, 24 h after cold exposure, which was followed by higher NO production and signaling, as confirmed by higher NO and 3-NT-modified proteins levels, respectively. Such increases in the NOS/NO pathway observed in the current study are concordant with previously shown increases in UCP1 expression in rpWAT during the first 7 days of cold acclimation [2], suggesting that NOS-mediated NO synthesis from l-arginine oxidation may mediate rpWAT browning in response to cold exposure.

This study further investigated whether l-arginine can increase NO synthesis in rpWAT at room temperature to the degree that mimics a cold-induced increase in NO synthesis and whether this increase translates into a cold-like browning phenotype of rpWAT. A wealth of literature has shown that l-arginine, when applied at physiological levels in rats, equivalent to a dose of 9 g/day of l-arginine in humans [37], increases the expression of NOS and fosters NO synthesis and subsequent signaling [9,13,28,38,39]. However, the efficacy of l-arginine is tissue-specific due to the differential presence of regulatory mechanisms acting on NO synthesis, metabolism, and bioavailability. Therefore, a comprehensive analysis of protein expression of eNOS, iNOS, and arginase I and II in concert with 3-NT-modified proteins and NO levels during 1-, 3-, and 7-day l-arginine treatment was done in rpWAT. The study revealed that l-arginine treatment increased iNOS, NO, and 3-NT-modified protein levels in rpWAT, despite both arginase I and II also being positively upregulated by l-arginine in rpWAT. Thereby, achieved levels of NO and associated downstream RNS-mediated signaling (evidenced by higher 3-NT-modified protein levels) corresponded to the cold-induced increases of these parameters. Therefore, the provision of 2.25% l-arginine for 7 days instigated similar increases in NO and RNS levels as those induced in response to cold exposure, indicating that l-arginine can be used to test cold-like induced NO signaling in rpWAT browning.

Several previous studies reported that long-term, 4–12 weeks, supplementation with l-arginine (1.51%) reduces white fat mass in genetically and diet-induced obese rats [18,20]. Besides, several clinical trials reported that l-arginine supplementation used alone or in combination with several other amino acids, a hypocaloric diet, or exercise could promote weight-reducing effects, primarily as a result of decreases in several anthropometric indices of abdominal obesity [21,22,25,27,29,40,41,42]. Accordingly, in the current animal study, we evaluated body weight gain, AI, and rpWAT mass as the indices of overall and visceral obesity, respectively. The results of the current study confirmed that a one-week-long treatment with 2.25% l-arginine suppressed body weight gain and decreased relative rpWAT mass. Lower serum leptin levels also corroborated fat reduction induced by l-arginine. Such results are in agreement with the results of Fu et al. [20], showing that 10 weeks of 1.51% l-arginine supplementation reduced rpWAT weight and serum leptin concentration, and indicate that even short-term l-arginine treatment, albeit in a higher dose (2.25%), exerts a significant fat mass depleting effect.

A decrease in body weight gain, in particular, a decrease in visceral adipose tissue mass, associated with l-arginine treatment, may improve the systemic lipid profile. Our results did not, however, confirm the significant differences in the triglycerides or cholesterol ratio, and the total, LDL, and HDL levels were higher in the l-arginine-treated group. Although there are currently no definitive conclusions for l-arginine supplementation on improving lipid profile [43], several experimental and clinical reports showed that l-arginine might exert positive effects on the lipid profile in rats and humans [44,45,46]. The discrepancies in our results and other findings may conceivably be attributed to the supplementation dosage and the differences in duration, as probably more time (4 weeks) is needed to detect changes in the systemic lipid status.

Hitherto, the effect of l-arginine on body fat reduction has been mainly ascribed to increased lipolysis in response to l-arginine-induced increase in energy metabolism in muscles and BAT [13,18,23,47]. In agreement, hydrolysis of triacylglycerols (as indicated by increased protein expression of HSL and ATGL) in rpWAT by l-arginine treatment may increase consequently to meet the energy requirements of BAT [9,48] and skeletal muscles [39,49], as shown in our previous studies. In addition, 10 weeks of l-arginine supplementation may increase the oxidative and antioxidative capacity of WAT by targeting the expression of several transcription factors and enzymes involved in the oxidative metabolism of glucose and antioxidative defense in WAT, like PGC-1α, AMP-activated protein kinase, and heme oxygenase-3, respectively [20]. PGC-1α is a leading instigator of mitochondrial biogenesis and oxidative metabolism, present and highly responsive in tissues with substantial lipid-oxidative capacity, but only ectopic in WAT. PGC-1α stimulates mitochondrial biogenesis and acts on NRF1 and PPARα expression, upregulating the expression of nuclear and mitochondrial genes that encode mitochondrial proteins and lipid-oxidizing enzymes [50,51]. PGC-1α also strongly coactivates PPARα, which increases UCP1 expression [52]. Here, we showed that PGC-α1 mRNA expression increased instantly, after 24 h of l-arginine treatment 5-fold, and this increase was maintained for 7 days of l-arginine treatment. This was followed by parallel increases in mitochondrial DNA content, PPARα (from 1st day onward), and transient increases in NRF1 and enzymes mediating mitochondrial fatty acid import and β-oxidation, CPT1 and ACADM, respectively, on days 1 and 3 of l-arginine treatment. All detected changes indicate the relevance of l-arginine-induced upregulation of PGC-1α in subsequent induction of fat oxidation capacity of rpWAT.

To relate such effects of l-arginine with browning phenotype and its relevance, we investigated protein expression and tissue distribution of UCP1 by Western blot and immunohistochemistry, as well as the genetic markers of thermogenic and beige adipocytes, *Cidea*, *Cd137*, and *Tmem26*. Upon cold exposure, most adipocytes within rpWAT underwent transient brown-fat-like thermogenic recruitment, with the most significant increases in UCP1 expression on days 3 and 7 of cold exposure [2]. Adipocytes retained their unilocular and paucilocular morphology on the tissue structure level, while thermogenic, polygonal, and multilocular beige cells subsequently appeared on persistent 45-day cold acclimation [2]. Here, we showed that, in a similar way, l-arginine increased UCP1 protein expression level after 3 days of treatment. Moreover, oddly to the appearance of unilocular UCP1-positive adipocytes on day 3 of cold exposure, on day 3 of l-arginine treatment, the increase in UCP1 expression was accompanied by the occurrence of islands of multilocular and polygonal cells highly positive for UCP1. Genetic markers of browning indicate a dual origin of UCP1-positive adipocytes, which may include a transient phenotype shift due to an increase in beige adipocyte-specific markers (*Cd137* and *Tmem26*), but does not exclude de novo occurrence of beige adipocytes due to an increase in nonspecific thermogenic (*Cidea*) and proliferation (PCNA) markers. Additional research is needed to investigate the mechanism of l-arginine action, i.e., the origin of UCP1-positive beige cells, both after short-term (7 days, as used in the current study) and long-term (≥4 weeks, as used in previous studies) l-arginine supplementation, as they may differ in respect to treatment duration and specific WAT depot. Further dissection of the effects of l-arginine on the molecular browning program in primary white adipocytes and preadipocytes in vitro from both visceral and subcutaneous WAT will provide more insights into mechanisms of WAT browning upon l-arginine treatment and its utility in the treatment of obesity and associated diseases. In addition, functional analysis and mitochondrial respiration studies will generate insightful data on the thermogenic effects of l-arginine on these adipocytes. Our efforts along these lines are in progress.

In conclusion, the most potent physiological stimulus for WAT browning—exposure to low temperature, acts by increasing the NO synthesis and subsequent nitrosative redox signaling. Treatment with 2.25% l-arginine at room temperature for the same period emulates cold-induced NO signaling. The resulting increase in NO associated with l-arginine treatment instigates the key structural, metabolic, and molecular biomarkers of WAT browning—gene and protein expression of key thermogenic and beige adipocyte genetic markers, transcription factors, and their downstream mitochondrial targets, including increased expression of UCP1 and the occurrence of UCP1-positive multilocular beige adipocytes. This switch in molecular signature from white- toward beige-like adipocytes results in rpWAT depot weight and body weight decreases at the end of the 7-day l-arginine treatment. The results suggest that l-arginine could be a good pharmacological alternative to cold exposure in the implementation of WAT browning in future anti-obesity strategies.

## Figures and Tables

**Table 1:** Effects of l-arginine on blood glucose and serum leptin, adiponectin, total, HDL-, LDL-cholesterol, and triglyceride levels; 2.5-month-old rats were randomly assigned to receive drinking water or 2.25% l-arginine-HCl in drinking water for 7 days.

	Control	l-Arginine
Glucose (mmol L^−1^)	5.8 ± 0.5	6.0 ± 0.4
Total cholesterol (mmol L^−1^)	1.63 ± 0.2	1.9 ± 0.2 *
LDL-cholesterol (mmol L^−1^)	0.51 ± 0.04	0.61 ± 0.06 *
HDL-cholesterol (mmol L^−1^)	0.7 ± 0.1	0.9 ± 0.1 *
Triglyceride (mmol L^−1^)	0.7 ± 0.2	0.8 ± 0.1
LDL-cholesterol/HDL-cholesterol	0.7 ± 0.07	0.7 ± 0.02
Total cholesterol/HDL-cholesterol	2.4 ± 0.2	2.3 ± 0.1
TG/HDL-cholesterol	0.97 ± 0.2	0.98 ± 0.03
Leptin (pg mL^−1^)	985.7 ± 90.0	664.8 ± 56.4 ***
Adiponectin (ng mL^−1^)	93.9 ± 1.9	88.9 ± 8.3

The results represent the means ± SD (*n* = 6 per group). * Comparison with control; * *p* < 0.05; *** *p* < 0.001.

## Data Availability

The data presented in this study are available on reasonable request from the corresponding author.

## Supplementary Materials

The following supporting information can be downloaded at: https://www.mdpi.com/article/10.3390/pharmaceutics14071368/s1, Figure S1: Western blot; Table S1: RT-PCR.

## References

[1] Sakers A., De Siqueira M.K., Seale P., Villanueva C.J. (2022). Adipose-tissue plasticity in health and disease. Cell.

[2] Jankovic A., Golic I., Markelic M., Stancic A., Otasevic V., Buzadzic B., Korac A., Korac B. (2015). Two key temporally distinguishable molecular and cellular components of white adipose tissue browning during cold acclimation. J. Physiol..

[3] Nedergaard J., Golozoubova V., Matthias A., Asadi A., Jacobsson A., Cannon B. (2001). UCP1: The only protein able to mediate adaptive non-shivering thermogenesis and metabolic inefficiency. Biochim. Biophys. Acta Bioenerg..

[4] Ricquier D. (2011). Uncoupling protein 1 of brown adipocytes, the only uncoupler: A historical perspective. Front. Endocrinol..

[5] Flachs P., Rossmeisl M., Kuda O., Kopecky J. (2013). Stimulation of mitochondrial oxidative capacity in white fat independent of UCP1: A key to lean phenotype. Biochim. Biophys. Acta Mol. Cell Biol. Lipids.

[6] Bartelt A., Bruns O.T., Reimer R., Hohenberg H., Ittrich H., Peldschus K., Kaul M.G., Tromsdorf U.I., Weller H., Waurisch C. (2011). Brown adipose tissue activity controls triglyceride clearance. Nat. Med..

[7] Yoneshiro T., Wang Q., Tajima K., Matsushita M., Maki H., Igarashi K., Dai Z., White P.J., McGarrah R.W., Ilkayeva O.R. (2019). BCAA catabolism in brown fat controls energy homeostasis through SLC25A44. Nature.

[8] Jankovic A., Otasevic V., Stancic A., Buzadzic B., Korac A., Korac B. (2017). Physiological regulation and metabolic role of browning in white adipose tissue. Horm. Mol. Biol. Clin. Investig..

[9] Petrović V., Buzadžić B., Korać A., Vasilijević A., Janković A., Korać B. (2010). NO modulates the molecular basis of rat interscapular brown adipose tissue thermogenesis. Comp. Biochem. Physiol. C Toxicol. Pharmacol..

[10] Kapur S., Picard F., Perreault M., Deshaies Y., Marette A. (2000). Nitric oxide: A new player in the modulation of energy metabolism. Int. J. Obes..

[11] Saha S.K., Kuroshima A. (2000). Nitric oxide and thermogenic function of brown adipose tissue in rats. Jpn. J. Physiol..

[12] Kikuchi-Utsumi K., Gao B., Ohinata H., Hashimoto M., Yamamoto N., Kuroshima A. (2002). Enhanced gene expression of endothelial nitric oxide synthase in brown adipose tissue during cold exposure. Am. J. Physiol. Regul. Integr. Comp. Physiol..

[13] Wu Z., Satterfield M.C., Bazer F.W., Wu G. (2012). Regulation of brown adipose tissue development and white fat reduction by L-arginine. Curr. Opin. Clin. Nutr. Metab. Care.

[14] Roberts L.D., Ashmore T., Kotwica A.O., Murfitt S.A., Fernandez B.O., Feelisch M., Murray A.J., Griffin J.L. (2015). Inorganic nitrate promotes the browning of white adipose tissue through the nitrate-nitrite-nitric oxide pathway. Diabetes.

[15] Peleli M., Ferreira D.M.S., Tarnawski L., McCann Haworth S., Xuechen L., Zhuge Z., Newton P.T., Massart J., Chagin A.S., Olofsson P.S. (2020). Dietary nitrate attenuates high-fat diet-induced obesity via mechanisms involving higher adipocyte respiration and alterations in inflammatory status. Redox Biol..

[16] Van Faassen E.E., Bahrami S., Feelisch M., Hogg N., Kelm M., Kim-Shapiro D.B., Kozlov A.V., Li H., Lundberg J.O., Mason R. (2009). Nitrite as regulator of hypoxic signaling in mammalian physiology. Med. Res. Rev..

[17] Hobbs A.J., Fukuto J.M., Ignarro L.J. (1994). Formation of free nitric oxide from L-arginine by nitric oxide synthase: Direct enhancement of generation by superoxide dismutase. Proc. Natl. Acad. Sci. USA.

[18] Jobgen W., Meininger C.J., Jobgen S.C., Li P., Lee M.J., Smith S.B., Spencer T.E., Fried S.K., Wu G. (2009). Dietary L-arginine supplementation reduces white fat gain and enhances skeletal muscle and brown fat masses in diet-induced obese rats. J. Nutr..

[19] Jankovic A., Korac A., Buzadzic B., Stancic A., Otasevic V., Ferdinandy P., Daiber A., Korac B. (2017). Targeting the NO/superoxide ratio in adipose tissue: Relevance to obesity and diabetes management. Br. J. Pharmacol..

[20] Fu W.J., Haynes T.E., Kohli R., Hu J., Shi W., Spencer T.E., Carroll R.J., Meininger C.J., Wu G. (2005). Dietary L-arginine supplementation reduces fat mass in Zucker diabetic fatty rats. J. Nutr..

[21] Monti L.D., Setola E., Lucotti P.C.G., Marrocco-Trischitta M.M., Comola M., Galluccio E., Poggi A., Mammì S., Catapano A.L., Comi G. (2012). Effect of a long-term oral l-arginine supplementation on glucose metabolism: A randomized, double-blind, placebo-controlled trial. Diabetes Obes. Metab..

[22] Lucotti P., Setola E., Monti L.D., Galluccio E., Costa S., Sandoli E.P., Fermo I., Rabaiotti G., Gatti R., Piatti P.M. (2006). Beneficial effects of a long-term oral L-arginine treatment added to a hypocaloric diet and exercise training program in obese, insulin-resistant type 2 diabetic patients. Am. J. Physiol. Endocrinol. Metab..

[23] McKnight J.R., Satterfield M.C., Jobgen W.S., Smith S.B., Spencer T.E., Meininger C.J., McNeal C.J., Wu G. (2010). Beneficial effects of L-arginine on reducing obesity: Potential mechanisms and important implications for human health. Amino Acids.

[24] Ueda K., Nakamura Y., Yamaguchi M., Mori T., Uchida M., Fujita S. (2016). Amino acid mixture enriched with arginine, alanine, and phenylalanine stimulates fat metabolism during exercise. Int. J. Sport Nutr. Exerc. Metab..

[25] Sasai H., Ueda K., Tsujimoto T., Kobayashi H., Sanbongi C., Ikegami S., Nakata Y. (2017). Dose-ranging pilot randomized trial of amino acid mixture combined with physical activity promotion for reducing abdominal fat in overweight adults. Diabetes Metab. Syndr. Obes. Targets Ther..

[26] Ueda K., Sanbongi C., Takai S., Ikegami S., Fujita S. (2017). Combination of aerobic exercise and an arginine, alanine, and phenylalanine mixture increases fat mobilization and ketone body synthesis. Biosci. Biotechnol. Biochem..

[27] Ueda K., Sasai H., Tsujimoto T., Sanbongi C., Ikegami S., Kobayashi H., Shioya N., Suzuki S., Nakata Y. (2018). Randomized trial of amino acid mixture combined with physical activity promotion for abdominal fat reduction in overweight adults. Diabetes Metab. Syndr. Obes. Targets Ther..

[28] Jobgen W.S., Fried S.K., Fu W.J., Meininger C.J., Wu G. (2006). Regulatory role for the arginine-nitric oxide pathway in metabolism of energy substrates. J. Nutr. Biochem..

[29] Hurt R.T., Ebbert J.O., Schroeder D.R., Croghan I.T., Bauer B.A., McClave S.A., Miles J.M., McClain C.J. (2014). L-arginine for the treatment of centrally obese subjects: A pilot study. J. Diet. Suppl..

[30] Lončar D. (1991). Convertible adipose tissue in mice. Cell Tissue Res..

[31] Giordano A., Morroni M., Carle F., Gesuita R., Marchesi G.F., Cinti S. (1998). Sensory nerves affect the recruitment and differentiation of rat periovarian brown adipocytes during cold acclimation. J. Cell Sci..

[32] Himms-Hagen J., Melnyk A., Zingaretti M.C., Ceresi E., Barbatelli G., Cinti S. (2000). Multilocular fat cells in WAT of CL-316243-treated rats derive directly from white adipocytes. Am. J. Physiol. Cell Physiol..

[33] Frontini A., Cinti S. (2010). Distribution and Development of Brown Adipocytes in the Murine and Human Adipose Organ. Cell Metab..

[34] Waldén T.B., Hansen I.R., Timmons J.A., Cannon B., Nedergaard J. (2012). Recruited vs. nonrecruited molecular signatures of brown, “brite,” and white adipose tissues. Am. J. Physiol. Endocrinol. Metab..

[35] Van Der Lans A.A.J.J., Hoeks J., Brans B., Vijgen G.H.E.J., Visser M.G.W., Vosselman M.J., Hansen J., Jörgensen J.A., Wu J., Mottaghy F.M. (2013). Cold acclimation recruits human brown fat and increases nonshivering thermogenesis. J. Clin. Invest..

[36] van Marken Lichtenbelt W.D., Vanhommerig J.W., Smulders N.M., Drossaerts J.M.A.F.L., Kemerink G.J., Bouvy N.D., Schrauwen P., Teule G.J.J. (2009). Cold-Activated Brown Adipose Tissue in Healthy Men. N. Engl. J. Med..

[37] Evans R.W., Fernstrom J.D., Thompson J., Morris S.M., Kuller L.H. (2004). Biochemical responses of healthy subjects during dietary supplementation with L-arginine. J. Nutr. Biochem..

[38] Petrović V., Buzadžić B., Korać A., Korać B. (2010). Antioxidative defense and mitochondrial thermogenic response in brown adipose tissue. Genes Nutr..

[39] Stancic A., Buzadzic B., Korac A., Otasevic V., Jankovic A., Vucetic M., Markelic M., Velickovic K., Golic I., Korac B. (2013). Regulatory role of PGC-1α/PPAR signaling in skeletal muscle metabolic recruitment during cold acclimation. J. Exp. Biol..

[40] Zeinali Khosroshahi M., Asbaghi O., Moradi S., Rezaei kelishadi M., Kaviani M., Mardani M., Jalili C. (2020). The effects of supplementation with L-arginine on anthropometric indices and body composition in overweight or obese subjects: A systematic review and meta-analysis. J. Funct. Foods.

[41] Mousavi S.M., Milajerdi A., Fatahi S., Rahmani J., Zarezadeh M., Ghaedi E., Varkaneh H.K. (2021). The effect of L-arginine supplementation on obesity-related indices: A systematic review and meta-analysis of randomized clinical trials. Int. J. Vitam. Nutr. Res..

[42] Alizadeh M., Daneghian S., Ghaffari A., Ostadrahimi A., Safaeiyan A., Estakhri R., Gargari B.P. (2010). The effect of hypocaloric diet enriched in legumes with or without L- Arginine and selenium on anthropometric measures in central obese women. J. Res. Med. Sci..

[43] Hadi A., Arab A., Moradi S., Pantovic A., Clark C.C.T., Ghaedi E. (2019). The effect of l-arginine supplementation on lipid profile: A systematic review and meta-analysis of randomised controlled trials. Br. J. Nutr..

[44] Dashtabi A., Mazloom Z., Fararouei M., Hejazi N. (2015). Oral L-Arginine Administration Improves Anthropometric and Biochemical Indices Associated With Cardiovascular Diseases in Obese Patients: A Randomized, Single Blind Placebo Controlled Clinical Trial. Res. Cardiovasc. Med..

[45] Míguez I., Mariño G., Rodríguez B., Taboada C. (2004). Effects of dietary L-arginine supplementation on serum lipids and intestinal enzyme activities in diabetic rats. J. Physiol. Biochem..

[46] Miczke A., Suliburska J., Pupek-Musialik D., Ostrowska L., Jabłecka A., Krejpcio Z., Skrypnik D., Bogdański P. (2015). Effect of L-arginine supplementation on insulin resistance and serum adiponectin concentration in rats with fat diet. Int. J. Clin. Exp. Med..

[47] Tan B., Li X., Yin Y., Wu Z., Liu C., Tekwe C.D., Wu G. (2012). Regulatory roles for L-arginine in reducing white adipose tissue. Front. Biosci..

[48] Vucetic M., Otasevic V., Korac A., Stancic A., Jankovic A., Markelic M., Golic I., Velickovic K., Buzadzic B., Korac B. (2011). Interscapular brown adipose tissue metabolic reprogramming during cold acclimation: Interplay of HIF-1α and AMPKα. Biochim. Biophys. Acta Gen. Subj..

[49] Stancic A., Filipovic M., Ivanovic-Burmazovic I., Masovic S., Jankovic A., Otasevic V., Korac A., Buzadzic B., Korac B. (2017). Early energy metabolism-related molecular events in skeletal muscle of diabetic rats: The effects of L-arginine and SOD mimic. Chem. Biol. Interact..

[50] Puigserver P., Wu Z., Park C.W., Graves R., Wright M., Spiegelman B.M. (1998). A cold-inducible coactivator of nuclear receptors linked to adaptive thermogenesis. Cell.

[51] Wu Z., Puigserver P., Andersson U., Zhang C., Adelmant G., Mootha V., Troy A., Cinti S., Lowell B., Scarpulla R.C. (1999). Mechanisms controlling mitochondrial biogenesis and respiration through the thermogenic coactivator PGC-1. Cell.

[52] Puigserver P., Spiegelman B.M. (2003). Peroxisome proliferator-activated receptor-γ coactivator 1α (PGC-1α): Transcriptional coactivator and metabolic regulator. Endocr. Rev..

