# Supplementary material for: l-Arginine Induces White Adipose Tissue Browning—A New Pharmaceutical Alternative to Cold
Source: Pharmaceutics. 2022 Jun 28;14(7):1368. doi: 10.3390/pharmaceutics14071368 (PMC9324995; doi:10.3390/pharmaceutics14071368)
Supplement: Supplementary file 1 [file pharmaceutics-14-01368-s001.zip › pharmaceutics-1757757-supplementary.pdf]

Western blot

Supplementary Figure S1. Images of whole blots against endothelial nitric oxide synthase (eNOS), inducible nitric oxide synthase (iNOS), 3-nitrotyrosine (3-NT), arginase I, arginase II, hormone sensitive lipase (HSL), adipose triglyceride lipase (ATGL), monoacyl-glycerol acyltransferase 1 (MGAT1), carnitine palmitoyltransferase 1 (CPT1), proliferating cell nuclear antigen (PCNA), nuclear factor erythroid 2-related factor 2 (Nrf2), uncoupling protein 1 (UCP1), nuclear respiratory factor 1 (NRF1), peroxisome proliferator-activated receptor-α (PPARα), acyL-CoA dehydrogenase medium chain (ACADM) and β-actin for retroperitoneal white adipose tissue (rpWAT). Each image is representative of three independent trials, showing one representative band per group (control, cold-exposed for 1 day, cold-exposed for 3 days, cold-exposed for 7 days, L-arginine treated for 1 day, L-arginine treated for 3 days, L-arginine treated for 7 days, respectively). Prior to loading, six samples from each group were pooled to obtain one sample shown in blots.

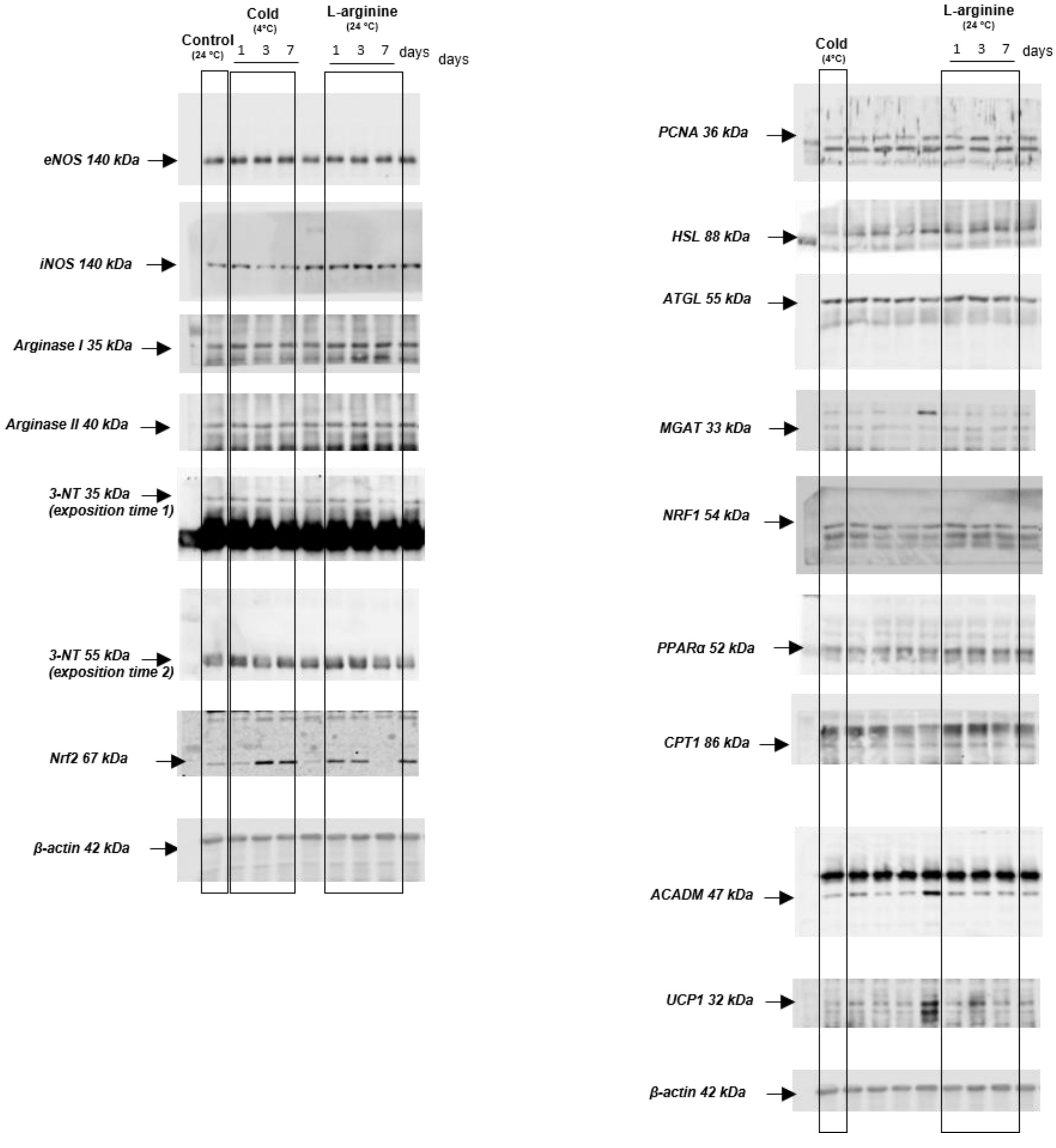

RT-PCR

Supplementary Table S1. Primers sequences and cycling conditions for RT-PCR.

Gene	Sequence	Cycle number
PGC-1α		
Forward:	5′ - TGC CCC TGC CAG TCA CAG GA - 3′	45
Reverse:	5′ - GCT CAG CCG AGG ACA CGA GG - 3′	
Cycle protocol:	15′′ at 95 ºC, 30′′ at 60 ºC, 30′′ at 72 ºC	
Cidea		
Forward:	5′ - TCA GAC CCT AAG AGA CAA CAC A - 3′	40
Reverse:	5′ - CAT TGA GAC AGC CGA GGA - 3′	
Cycle protocol:	15′′ at 95 ºC, 30′′ at 60 ºC, 20′′ at 72 ºC	
Cd137		
Forward:	5′ - AAG CAA CCA TTT AAG AAG GCG G - 3′	40
Reverse:	5′ - GGA TGG CAC ATT ACA GCT CG - 3′	
Cycle protocol:	15′′ at 95 ºC, 30′′ at 60 ºC, 20′′ at 72 ºC	
Tmem26		
Forward:	5′ - GCT GTT CCT GTT GCA TTC CC - 3′	40
Reverse:	5′ - GGA GAA AGC CAT TTG TAG CCT C - 3′	
Cycle protocol:	15′′ at 95 ºC, 30′′ at 60 ºC, 20′′ at 72 ºC	
18 rRNK		
Forward:	5′ - CAC GGA CAG GAT TGA CAG AT - 3′	
Reverse:	5′ - CAA ATC GCT CCA CCA ACT AA - 3′	
